# Feature Selection Based on Machine Learning in MRIs for Hippocampal Segmentation

**DOI:** 10.1155/2015/814104

**Published:** 2015-05-18

**Authors:** Sabina Tangaro, Nicola Amoroso, Massimo Brescia, Stefano Cavuoti, Andrea Chincarini, Rosangela Errico, Paolo Inglese, Giuseppe Longo, Rosalia Maglietta, Andrea Tateo, Giuseppe Riccio, Roberto Bellotti

**Affiliations:** ^1^Istituto Nazionale di Fisica Nucleare, Sezione di Bari, Via Orabona 4, 70125 Bari, Italy; ^2^Dipartimento Interateneo di Fisica, Università degli Studi di Bari, Via Amendola 173, 70126 Bari, Italy; ^3^Astronomical Observatory of Capodimonte, INAF, Salita Moiariello 16, 80131 Napoli, Italy; ^4^Istituto Nazionale di Fisica Nucleare, Sezione di Genova, Via Dodecaneso 33, 16146 Genova, Italy; ^5^Dipartimento Interateneo di Fisica, Università degli Studi di Genova, Via Dodecaneso 33, 16146 Genova, Italy; ^6^Dipartimento di Fisica, Università degli Studi di Napoli Federico II, Complesso Universitario di Monte Sant'Angelo, Via Cintia 1, 80126 Napoli, Italy; ^7^Istituto di Studi sui Sistemi Intelligenti per l'Automazione, CNR, Via Giovanni Amendola 122/D-I, 70126 Bari, Italy

## Abstract

Neurodegenerative diseases are frequently associated with structural changes in the brain. Magnetic resonance imaging (MRI) scans can show these variations and therefore can be used as a supportive feature for a number of neurodegenerative diseases. The hippocampus has been known to be a biomarker for Alzheimer disease and other neurological and psychiatric diseases. However, it requires accurate, robust, and reproducible delineation of hippocampal structures. Fully automatic methods are usually the voxel based approach; for each voxel a number of local features were calculated. In this paper, we compared four different techniques for feature selection from a set of 315 features extracted for each voxel: (i) filter method based on the Kolmogorov-Smirnov test; two wrapper methods, respectively, (ii) sequential forward selection and (iii) sequential backward elimination; and (iv) embedded method based on the Random Forest Classifier on a set of 10 T1-weighted brain MRIs and tested on an independent set of 25 subjects. The resulting segmentations were compared with manual reference labelling. By using only 23 feature for each voxel (sequential backward elimination) we obtained comparable state-of-the-art performances with respect to the standard tool FreeSurfer.

## 1. Introduction

The analysis of medical images such as magnetic resonance images (MRIs) is useful to investigate and identify the structural alterations in the brain, frequently associated with dementia or neurodegenerative diseases. In this context, the hippocampal segmentation is used to study and detect the correlation between the morphological anomalies of the hippocampus and the occurrence of the Alzheimer's disease. Hence its importance is strictly related to the early prediction of the dementia [1, 2]. Since the manual tracing is time-consuming and highly operator-dependent, it is important to make this process as much automatic as possible.

As discussed in [3], automatic image analysis and classification methods exist, which are able to recognize brain anomalies at the level of the single patient, which is more useful than at the level of groups or categories of individuals. Nonetheless they potentially require a large amount of parameters (vector of features) to properly manage all differences and specific features of the human brain among individuals, causing the parameter space to explode in terms of complexity, redundancy, and noise. To find a limited amount of features able to recognize patterns with a sufficient level of accuracy and without requiring a huge computational effort, would be indeed very helpful. This is especially true when the feature selection and classification are performed by machine learning techniques, since the intrinsic self-organizing selection of important features and their cross-correlation remove any potential biased interpretability of the feature space.

Several approaches have been proposed to reach different levels of automation [4]. Among known methods, we quote just Morra et al. [5, 6], which suggest different automatic methods based on support vector machines (SVM) and hierarchical Adaboost, by considering about 18,000 voxel features, and FreeSurfer [7], a standard medical software tool for the analysis of cortical and subcortical anatomy, which performs a segmentation on cortical surface streams by constructing models of boundaries among white and gray matter.

Similarly, for an automatic hippocampal segmentation, we use a voxel-based approach by using 315 local features for each voxel included in a parahippocampal region larger than the hippocampus volume. Extracting 315 features for such a large number of voxels needs massive processing time and massive computational resources. For this reason, we consider crucial the issue of feature selection (FS) or reduction. The utility of feature selection is (a) to avoid overfitting, by minimizing the dimension of the parameter space and improving model performance, that is, prediction performance in the case of supervised classification and better cluster detection in the case of clustering, (b) to provide faster and more cost-effective models, (c) to gain a deeper insight into the underlying processes that generated the data, and (d) to optimize the processing time and massive computational resource.

There is a price to be paid for this advantage. To search for a subset of relevant features introduces in fact an additional layer of complexity in the modeling task: it needs to find the optimal model parameters for the optimal feature subset, as there is no guarantee that the optimal parameters for the full input feature set are equally optimal also for the best feature subset [8, 9].

By providing a small quantity of features, it may reduce the computational time as being proportional to the number of features. Furthermore, in some cases it allows to gain a better classification accuracy [10]. Also, the reduction of the feature's number is necessary when, to train the classifier, only a limited number of examples is available. In this regard, it is shown that, for the same error rate, a classifier requires a training whose duration grows exponentially with the number of variables [11–13].

Feature reduction, therefore, includes any algorithm that finds a subset of input feature set. A feature reduction capability is present also in more general methods based on transformations or combinations of the input feature set (feature extraction algorithms). An example being the well-known principal component analysis (PCA), which eliminates the redundancy of information by generating new features set by a combination of input features [14].

However, the best feature selection, by preserving the original semantics of features, permits also to maintain a coherent interpretability. The main goal of this study is to exemplify and demonstrate the benefits of applying FS algorithms in hippocampus segmentation field.

## 2. Materials

The database used to perform the described experiments is composed by thirty-five T1-weighted whole brain MR images and the corresponding manually segmented bilateral hippocampi (masks). All images were acquired on a 1.0 *T* scanner according to MP-RAGE sequence for magnetic resonance imaging of the brain [15–17].

The images are derived from the Open Access Series of Imaging Studies (OASIS). In particular we used 35 MP-RAGE MRI brain scans with a resolution of 1 mm^3^ provided in occasion of the MICCAI SATA challenge workshop 2013 [18]. By using this homogeneous data sample it was possible to reduce the training image subsample without loss of generality and learning capabilities, giving the possibility to keep a sufficiently wide test set to perform a well-posed statistical analysis on the feature selection performances.

The image processing and classification were carried out blindly with respect to the subject status.

The first stage of our analysis chain requires an image preprocessing to standardize them both spatially and in gray intensity. This operation is obtained by registering the images on the Montreal Neurological Institute (MNI) standard template (ICBM152) using 12-parameter affine-registration and subsequent resampling on an isotropic grid with 1 mm^3^ voxel size.

In order to reduce the computational time of the analysis, from the MRI, spatially standardized, two volumes containing the left and right hippocampus including the relevant parahippocampal regions are extracted using a new method FAPoD (fully automatic algorithm based on point distribution model) described in [19, 20].

We can then proceed with the feature extraction only in this identified region of interest: we approach a binary classification voxel-based problem, where the categories are* hippocampus* or* not-hippocampus*, that is, based on supervised pattern recognition systems. The features should contain information relevant to the classification task. Since manual segmentation of the hippocampus is based on local texture information, we adopted the related features. In the analysis presented here for each voxel a vector whose elements represent information about position, intensity, neighboring texture [21], and local filters was obtained.

Texture information was expressed using both Haar-like and Haralick features [6, 22].

The Haralick features were calculated from the normalized gray level cooccurrence matrices (GLCM) created on the *m* × *m* voxels projection subimages of the volume of interest; *m* defines the size of overlapping sliding-windows. For each voxel, values of *m* varying from 3 to 9 were used. Each element (*k*, *p*) of a cooccurrence matrix indicates the probability that two voxels, separated by a specified spatial angle and distance, have gray levels *k* and *p*, respectively.

A subset of Haralick features is sufficient to obtain a satisfactory discrimination. To establish which of the original 14 GLCM Haralick features gives the best recognition rate, several preliminary recognition experiments were carried out [23]. The resulting best configuration has been individuated in 4 features: energy, contrast, correlation, and inverse difference moment [20].

Finally, the gradients calculated in different directions and at different distances were included as additional features. The best analysis configuration, expressed by the highest values of statistical indicators (see Section 3), was obtained with 315 features, described in Table 1.

By summarizing, the knowledge base (KB) consisted of 35 regions of interest (ROI) extracted from as many images, each one composed of 7910 voxels, where each voxel is represented through a vector of 315 features. Therefore, the training set, including 10 randomly selected images, was formed by a total of 79100 × 315 entries. In quantitative terms, it can be considered a sufficiently wide dataset, qualitatively able to cover all feature types needed to perform a complete training, avoiding the useless redundancy of information not needed by machine learning methods [24] and leaving a sufficiently large amount of samples to be dedicated to the test sessions.

## 3. Methods

The FS techniques are usually counted in three categories, based on their internal combination between the selection and classification of the reduced parameter space. These categories are, respectively, named as wrapper, filter, and embedded methods [25].

*Filter* method is a technique based on the measurement of the importance of each single feature of the given parameter space [26]. The selected features are the most relevant to obtain a correct classification. This technique includes methods suitable for high-dimensional datasets, since they are computationally fast. Furthermore, they are independent from the classification algorithm and therefore their results can be used for all types of classifier. However, since each feature is considered separately from the others, their positive contribution based on the combined effect is neglected. The filter method used in our analysis is based on the Kolmogorov-Smirnov (K-S) test.

*Wrapper* methods basically integrate the two aspects of the workflow, that is, the model hypothesis and feature search [27]. This procedure involves the generation and evaluation of various subsets of features. Every generated feature subset is associated to a classification criterion (hence the name* wrapper*). Since the number of all possible feature subsets grows exponentially with the size of the dataset, some search heuristics can be adopted to reduce drastically the number of operations. They can be grouped into* deterministic* and* randomized* search methods. The advantage of these methods is the intrinsic best interaction among selected features and their classifiers, but with the downside of having a high computational cost and the risk of overfitting. The wrapper methods used in our analysis are, respectively, sequential forward selection (SFS) and sequential backward elimination (SBE).

Finally, in* embedded* methods the optimal feature subset search is directly nested into the classifier algorithm [28]. Such techniques can be interpreted in terms of a search within a combined parameter space, by mixing features and hypotheses. Analogously to wrapper methods, they include the interaction with classification algorithm but in a faster way. The embedded method used in our analysis is based on the Random Forest Classifier.

To recap, in our FS analysis we used the following:

- univariate filter method: Kolmogorov-Smirnov,
- deterministic wrapper methods: sequential forward selection (SFS) and sequential backward elimination (SBE),
- embedded method: Random Forest.

In addition, we have also used the PCA [29], being one of the most widely adopted feature reduction techniques, for comparison.

To estimate the goodness of the selected feature group we used the Näive Bayes Classifier [30], based on the simplified hypothesis that all attributes describing a specific instance on data are conditionally independent among themselves.

The FS analysis was performed in the 5-fold cross validation on 10 of 35 images in the database. The goodness of the selected group was tested on the remaining 25 images. As already discussed in Section 2, the selected training and test rates were considered sufficiently wide to ensure a well-posed training and the postprocessing statistical evaluation.

The *k*-fold cross validation is a technique able to avoid overfitting on data and is able to improve the generalization performance of the machine learning model. In this way, validation can be implicitly performed during training, by enabling at setup the standard leave-one-out *k*-fold cross validation mechanism [31]. The automatized process of the cross validation consists in performing *k* different training runs with the following procedure: (i) splitting of the training set into *k* random subsets, each one composed by the same percentage of the data set (depending on the *k* choice); (ii) at each run the remaining part of the data set is used for training and the excluded percentage for validation. While avoiding overfitting, the *k*-fold cross validation leads to an increase of the execution time estimable around *k* − 1 times the total number of runs.

Furthermore, the combination of the Bayes rule with the above simplified assumption has a positive impact on the model complexity and its computational time. In particular, the latter property pushed us to choose this model as embedded classifier for the feature selection problem.

The agreement between an automated segmentation estimate and a manual segmentation can be assessed using overlap measures. A number of measures are available: (a) Dice index [20, 32]; (b) efficiency; (c) purity of a class; (d) completeness of a class; (e) contamination of a class.

At the base of the statistical indicators adopted, there is the commonly known* confusion matrix*, which can be used to easily visualize the classification performance [33]: each column of the matrix represents the instances in a predicted class, while each row represents the instances in the real class. One benefit of a confusion matrix is the simple way in which it allows seeing whether the system is mixing different classes or not.

We remark here that we were mostly interested in the feature analysis related to the classification of the* hippocampus* class voxels. Therefore, we considered as particularly relevant the Dice index, usually referred to as the* true positive* class (*N*_*AA*_ in our confusion matrix), which in our case corresponds properly to* hippocampus* class. Since, by definition, the Dice index does not take the true negative rate into account, the rate of* not-hippocampus* voxels is not involved within this indicator. A statistical evaluation of this latter class, corresponding to the background voxels, has been primarily included for completeness and for coherency with the full confusion matrix representation. The highest relevance given to the* hippocampus* class analysis represents also a common evaluation criterion in such context [6].

In terms of binary classification, we were more interested to perform a feature selection analysis, rather than to improve the classification performances. Therefore, we imposed a standard classification threshold to 0.5 at the beginning of the experiments and maintained unchanged all over the entire described process, by considering it as sufficient for our specific purposes.

More specifically, for a generic two-class confusion matrix, we consider

(1) OUTPUT−Class  AClass  BTARGETClass  ANAANABClass  BNBANBB

we then use its entries to define the following statistical quantities.

*(i) Total Efficiency. *te is defined as the ratio between the number of correctly classified objects and the total number of objects in the data set. In our confusion matrix example it would be

(2) $$\begin{array}{c} \text{te}=\frac{N_{AA}+N_{BB}}{N_{AA}+N_{AB}+N_{BA}+N_{BB}}. \end{array}$$

*(ii) Purity of a Class. *pc*N* is defined as the ratio between the number of correctly classified objects of a class and the number of objects classified in that class. In our confusion matrix example it would be

(3) $$\begin{array}{c} \text{pc}A=\frac{N_{AA}}{N_{AA}+N_{BA}},\\ \text{pc}B=\frac{N_{BB}}{N_{AB}+N_{BB}}. \end{array}$$

*(iii) Completeness of a Class. *cmp*N* is defined as the ratio between the number of correctly classified objects in that class and the total number of objects of that class in the data set. In our confusion matrix example it would be

(4) $$\begin{array}{c} \text{cmp}A=\frac{N_{AA}}{N_{AA}+N_{AB}},\\ \text{cmp}B=\frac{N_{BB}}{N_{BA}+N_{BB}}. \end{array}$$

*(iv) Contamination of a Class. *cnt*N* is the dual of the purity; namely, it is the ratio between the misclassified objects in a class and the number of objects classified in that class; in our confusion matrix it example will be

(5) $$\begin{array}{c} \text{cnt}A=1-\text{pc}A=\frac{N_{BA}}{N_{AA}+N_{BA}},\\ \text{cnt}B=1-\text{pc}B=\frac{N_{AB}}{N_{AB}+N_{BB}}. \end{array}$$

*(v) Dice Index. *Dice, known also with the name of *F*_1_ *score*, is a frequent measure used in binary classification, which could be considered as a weighted average of the purity and completeness, reaching its best value at 1 and the worst at 0. By referring to our notation, we have the Dice defined as

(6) $$\begin{array}{c} \text{Dice}=2\cdot \frac{\text{pc}A*\text{cmp}A}{\text{pc}A+\text{cmp}A}=2\cdot \frac{N_{AA}}{2N_{AA}+N_{BA}+N_{AB}}. \end{array}$$

## 4. Results

By using Näive Bayes Classifier on all 315 input features, the goodness is estimated in 5-fold cross validation on 10 images. The results in terms of the statistics, derived from the confusion matrix, are shown in Table 2 and the Dice index is 0.60 ± 0.04.

The PCA applied to 315 input features returns the principal components (PCs) ordered by the amount of information they convey. The percentage of information contained in the first 98 PCs and in the first 197 PCs are, respectively, 90% and 99%.

Since our goal was to reduce the feature retaining the goodness in the classification, we considered the first 197 PCs containing 99.0% of the information. The results obtained are shown in Table 3 and the Dice index is 0.62 ± 0.07. As mentioned above, we used the Näive Bayes Classifier in 5-fold cross validation.

Compared to the use of all 315 original features, the values obtained with 197 PCs are on average 6% points lower in terms of Dice index. Therefore, to avoid loss of information, we considered all 315 PCs. The results are reported in Table 4 and the Dice index is 0.63 ± 0.03.

Even using all the PCs, the result was 5% points lower in terms of Dice Iindex. This result confirms what was already found by Golland et al. in [34]; that is, the selection of large-variance features performed by the PCA is not specifically suited for segmentation problems.

### 4.1. Kolmogorov-Smirnov Analysis

The K-S test provides an estimate of how much two distributions are related to each other. The K-S test allowed us to select only the features which have a correlation between the two* hippocampus* and* not-hippocampus* classes less than 5%, resulting in a total of 57 features.

As mentioned above, we used the Näive Bayes Classifier in 5-fold cross validation. The results obtained are shown in Table 5 and the Dice index is 0.67 ± 0.04.

The K-S test results are comparable with the original parameters space based on 315 features.

### 4.2. Sequential Forward Selection and Backward Elimination

**Figure 1:**
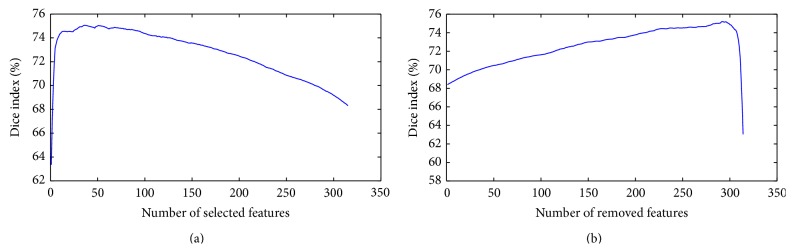
Best dice index of all the possible combinations of the relevant step in (a) sequential forward selection and in (b) sequential backward elimination methods.

The two FS methods belonging to the wrapper category experimented in our case were SFS and SBE. In Figure 1(a) on the ordinate axis, the top value of Dice index achieved between all possible combinations related to the reference step depicted on the horizontal axis is shown. At each step, the feature achieving the best performance is chosen, when used in combination with the selected features in the previous step. The step number coincides with the number of selected features (SFS).

In Figure 1(b) on the ordinate axis, the top value of Dice index achieved between all possible combinations related to the reference step depicted on the horizontal axis is shown. At each step the feature without which the best performances are obtained is removed. The step number coincides with the number of eliminated features (SBE).

**Figure 2:**
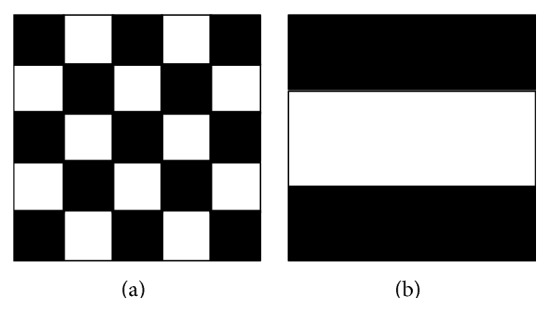
Haar-like template types 1 (a) and 2 (b) used in the experiments.

**Figure 3:**
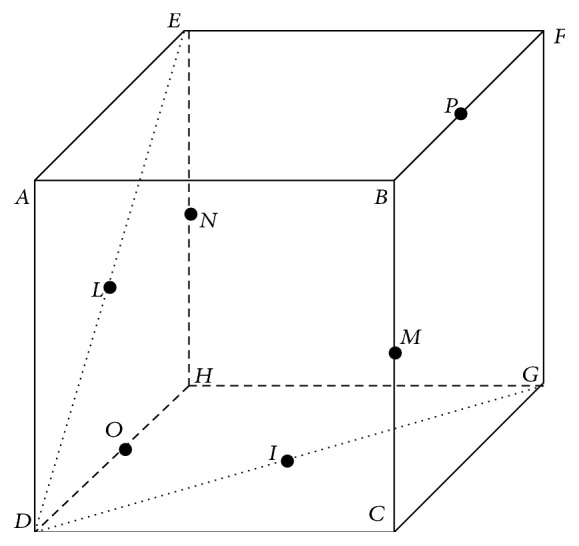
Representation of a generic cubic mask used for calculating the gradient features. The labeled points are either the vertexes of the cube or the median points of the segments.

We observe that the SFS method reaches its highest Dice index, 0.75, at step 36. So it means that the best performance, using the Näive Bayes Classifier, is obtained with only 36 selected features, listed in Table 7. In Figure 2 and Figure 3 a more detailed description of some features is shown.

The SBE method obtains its highest Dice index 0.75 at the step 292. Therefore, the best performance, evaluated with the Näive Bayes Classifier, is obtained by using the remaining 23 features (i.e., 315 − 292), listed in Table 9.

Tables 6 (with related Dice index is 0.75 ± 0.03) and 8 (with related Dice index is 0.75 ± 0.02), respectively, show the relative performance of the peak value in Figure 1.

### 4.3. Random Forest Analysis

The Random Forest classification methodology allowed us to estimate the feature importance [35]. To select the best subset we have performed a study of classification with cross validation procedure based on the Näive Bayes Classifier, varying the threshold on the feature importance index. The optimal threshold was related to the maximum Dice Index value and achieved with 222 features. Also in this case we used the Näive Bayes Classifier in 5-fold cross validation to evaluate the features selected by the Random Forest. The result obtained is shown in Table 10 and the Dice index is 0.69 ± 0.04.

### 4.4. Random Selection Test

**Figure 4:**
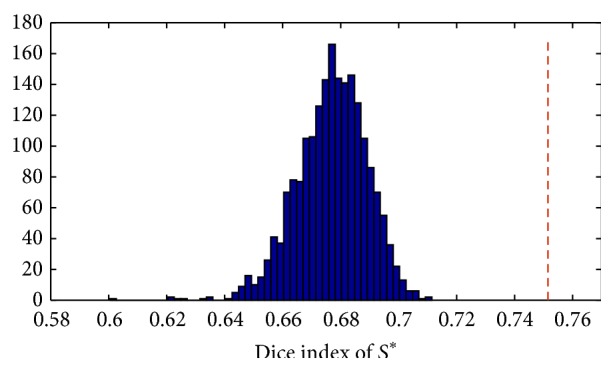
Distribution of 2000 random Dice values compared with true Dice (shown with the dashed red line) concerning 36 features obtained by the sequential forward selection.

Furthermore, we performed an additional group of tests to evaluate whether randomly selected samples of 36 features among the original 315 might lead to Dice indexes greater than or comparable with the Dice value obtained with SFS (0.75). To do so, we estimate the empirical probability density function of Dice under the null hypothesis that any set *S* ^*^ of 36 features provides a Dice value greater than or equal to the true Dice in predicting whether a voxel belongs to hippocampus or not. To test this hypothesis, 2000 sets *S* ^*^ were generated, each composed of 36 features randomly drawn from the ones available and the corresponding Dice values were evaluated. The obtained results are shown in Figure 4.

## 5. Discussion and Conclusion

The main goal of this work was to verify the possibility to reduce the number of required voxel features without losing or better by enhancing the classification performances. Moreover the reduction of the number of voxel features could also improve the computational efficiency of the classification.

As clearly resulting from a recent review, [3], by now the feature selection has to be considered as an essential step within the field of neuroimaging approached by the machine learning paradigm. Its importance is also invariant to the specific technique used to extract and codify the features from MRIs regions of interest, whether it is based on standard *n*-dimensional feature vectors or on pairwise dissimilarity representation. In the present work we investigated the application of several feature selection methods.

**Figure 5:**
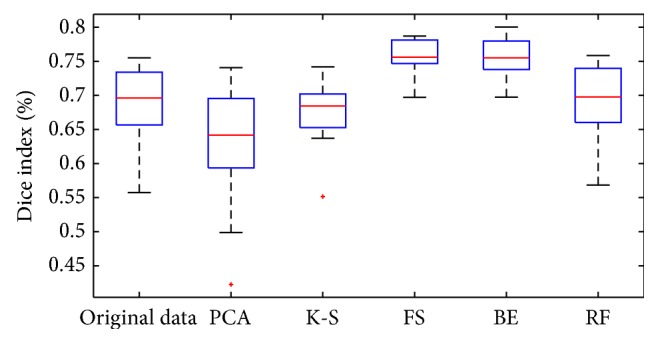
Dice index comparison for the following methods: original dataset (315 for each voxel); PCA (197 selected features); K-S test (57 selected features); SFS (36 selected features); SBE (23 selected features); Random Forest (222 selected features). Boxes have lines at the lower quartile, median, and upper quartile values, with whiskers extending to 1.5 times the interquartile range. Outliers are indicated by a plus sign.

The results obtained using different approaches are summarized in Table 11 and in Figure 5. We observe that by using these two selected subsets it is possible to obtain higher performances than using the entire input dataset.

By considering the percentage of random Dice values bigger than the best one with respect to the total number of random extractions, such value is zero. But, as it can be seen in Figure 4, in many cases it appears to obtain better performances by randomly extracting the feature sample rather than considering the complete set of 315 features.

Among the FS approaches presented in this work, the SFS and SBE show better performances.

We would underline that the results shown in Figure 5 have to be mainly interpreted as a comparison among the different methods of feature selection. What has to be stressed is that the performances are influenced by the feature information content and the image enhancement techniques employed. A quite simple method, such as the Näive Bayes Classifier, is able to reach state-of-the-art performances when preceded by a selection analysis on the feature space. A more detailed study of the classification methods and of the postprocessing technique which can be used to improve performances are presented in other studies [36, 37].

To test the goodness of the best feature selection methods presented in this paper we used the two selected sets formed, respectively, by 36 and 23 features on a blind test database composed of 25 MRIs (i.e., not used in training phase), in the algorithm cited in [36] (see Tables 7 and 9, resp.).

By analyzing the two subsets of selected features, it was obtained that 13 of the 23 extracted by the SBE method are also present in the sample of 36 features obtained by the SFS technique. Most of them are Haralick and Statistical features, except for the positional and Haar-like features, confirming the importance given by Haralick and Statistical types and a very low contribution of Haar-like type.

We remark that, by minimizing the presence of Haralick features, in particular the correlations, it allows improving the processing time and a better handling of the information content. In fact, among the three categories of features considered here, the Haralick type was the most* time-consuming* from the computational point of view.

The comparison of our FS methods with the widely used PCA demonstrates the very low performance of the PCA technique (as shown in Figure 5). This result is in agreement with the well-known downside of the method in presence of a very high nonlinearity of the feature correlations. It is also an indirect confirmation about the intrinsic difficulty to separate the* hippocampus* versus* not-hippocampus* classes from MRI images.

We conclude that the SFS and SBE techniques are two promising methods allowing to reduce the input space size, with a very low loss of information, and permitting classification performances comparable or even better than the case with a larger amount of features.

In fact, in terms of feature space dimension comparison, Morra et al. [6] performs a voxel-based segmentation using about 18,000 features with the weighted voting method AdaBoost [38] tested on a different image data set. In addition, FreeSurfer [7], which is a not voxel-based method considered as standard benchmark for MRI segmentation experiments, reaches a Dice value of 0.76 ± 0.05.

In this work, we observed that the selected features from both SFS and SBE methods are related to the high frequency component of the image. So this result would suggest which kind of features are best suitable for high frequency classification problems such as edge recognition. In fact, these correlation features, being based on intensity differences, are able to capture local information based on discontinuity rather than similarity.

Besides, this result is a further suggestion for a future investigation which is to put in practice a preprocessing procedure to enhance the contours of the structures contained in the image and to assess the usefulness of these procedures in the diagnosis support systems.

## Figures and Tables

**Table 1:** The 315 features extracted from the 3D MRI images. Of each group of 66 Haralick features, 13 are the gradients along the 13 diagonals, 5 are the principal moments, and the rest are the three sets of 16 textural features, one set for each plane of the voxels. The gradients for each voxel are measured in all directions at one voxel distance and the relative 3D positions are included as features.

Number	Description
1	Position
1	Grey level
66	Haralick features for mask 3 × 3
66	Haralick features for mask 5 × 5
66	Haralick features for mask 7 × 7
66	Haralick features for mask 9 × 9
49	Haar-like 3D features

**Table 2:** Classification result on all 315 input features using Näive Bayes Classifier in 5-fold cross validation based on confusion matrix.

315 input features	Completeness of a class	Purity of a class	Contamination of a class
Hippocampus	79%	62%	38%
Not-hippocampus	63%	80%	20%
Efficiency	**70% **		

**Table 3:** Classification result on the first 197 PCs using Näive Bayes Classifier using in 5-fold cross validation based on confusion matrix.

197 PCs	Completeness of a class	Purity of a class	Contamination of a class
Hippocampus	60%	68%	32%
Not-hippocampus	78%	72%	28%
Efficiency	**71% **		

**Table 4:** Classification result on all 315 PCs using Näive Bayes Classifier using in 5-fold cross validation based on confusion matrix.

315 PCs	Completeness of a class	Purity of a class	Contamination of a class
Hippocampus	86%	51%	49%
Not-hippocampus	36%	78%	22%
Efficiency	**58% **		

**Table 5:** Classification result on 57 features selected through Kolmogorov-Smirnov test using Näive Bayes Classifier using in 5-fold cross validation based on confusion matrix.

57 features Kolmogorov-Smirnov test	Completeness of a class	Purity of a class	Contamination of a class
Hippocampus	84%	57%	43%
Not-hippocampus	52%	81%	19%
Efficiency	** 66% **		

**Table 6:** Classification result on 36 features selected through forward selection method using Näive Bayes Classifier in 5-fold cross validation based on confusion matrix.

36 features Forward selection	Completeness of a class	Purity of a class	Contamination of a class
Hippocampus	82%	70%	30%
Not-hippocampus	73%	84%	16%
Efficiency	**77% **		

**Table 7:** Details of the 36 features resulting by the forward selection method using Näive Bayes Classifier.

36 features Forward selection	Haralick features			Haar-like features	Statistical features	
	Orientation	Coordinate	Mask size	Type	Mask size	Entry
Contrast^*^	135	*Y *	3			
Gradient^*^					5	$\bar{EC}$
Correlation	135	*X *	3			
Position^*^						Coordinates
Normalized gray level^*^						Value
Correlation^*^	45	*X *	5			
Gradient^*^					5	$\bar{DF}$
Correlation^*^	90	*Y *	9			
Correlation	45	*Y *	7			
Skewness^*^					7	
Homogeneity^*^	90	*X *	9			
Correlation	0	*Y *	5			
Correlation	90	*Z *	5			
Correlation^*^	45	*X *	3			
Correlation	135	*Z *	9			
Correlation	90	*Y *	5			
Correlation	135	*Z *	5			
Correlation	0	*Z *	7			
Correlation	90	*Z *	7			
Correlation	90	*Z *	9			
Correlation	0	*Y *	3			
Correlation	135	*X *	3			
Correlation	0	*Z *	9			
Template^*^				1		
Skewness^*^					5	
Correlation	90	*Z *	3			
Correlation	45	*X *	5			
Gradient					3	$\bar{MN}$
Template				2		
Correlation^*^	45	*X *	9			
Correlation	45	*Y *	5			
Correlation	90	*Y *	7			
Correlation	45	*Z *	5			
Gradient					9	$\bar{DF}$
Homogeneity	0	*Z *	9			
Correlation	0	*Y *	9			

The asterisk indicates the entries also present in the list of 23 SBE features. For Haralick features, the orientation in degrees, reference coordinate, and the size of the cubic mask used are reported. In case of Haar-like features, the entry value indicates the template type used (see Figure 2). For statistical/positional kind the size of the cubic mask used or the self-explained value is lister, depending on the specific feature type. In particular for gradients, the column named Entry indicates the segment of the reference diagonal as shown in Figure 3. All the features are listed in top-down order of their inclusion during the SFS procedure execution.

**Table 8:** Classification result on 23 features selected through backward elimination method using Näive Bayes Classifier in 5-fold cross validation based on confusion matrix.

23 features Backward elimination	Completeness of a class	Purity of a class	Contamination of a class
Hippocampus	83%	70%	30%
Not-hippocampus	73%	85%	15%
Efficiency	**77% **		

**Table 9:** Details of the 23 features resulting by the backward elimination method using Näive Bayes Classifier.

23 features Backward elimination	Haralick features			Haar-like features	Statistical features	
	Orientation	Coordinate	Mask size	Type	Mask size	Entry
Position^*^						Coordinates
Normalized gray level^*^						Value
Correlation	0	*Y *	7			
Correlation^*^	45	*X *	3			
Correlation^*^	45	*X *	5			
Correlation	45	*X *	7			
Correlation^*^	45	*X *	9			
Correlation	45	*Y *	9			
Correlation	45	*Y *	5			
Correlation^*^	90	*Y *	9			
Homogeneity	135	*Z *	3			
Gradient^*^					5	$\bar{DF}$
Gradient					7	$\bar{DF}$
Contrast^*^	135	*Y *	3			
Gradient					9	$\bar{OP}$
Homogeneity^*^	90	*X *	9			
Gradient					3	$\bar{BH}$
Skewness^*^					7	
Gradient^*^					5	$\bar{EC}$
Gradient					3	$\bar{IL}$
Template^*^				1		
Skewness^*^					5	
Gradient					5	$\bar{MN}$

The asterisk indicates the entries also present in the list of 36 SFS features. For Haralick features, the orientation in degrees, reference coordinate, and the size of the cubic mask used are reported. In case of Haar-like features, the entry value indicates the template type used (see Figure 2). For statistical/positional kind, the size of the cubic mask used and/or the self-explained value is listed, depending on the specific feature type. In particular for gradients, the column named Entry indicates the segment of the reference diagonal as shown in Figure 3.

**Table 10:** Classification result on 222 features selected through Random Forest method using Näive Bayes Classifier in 5-fold cross validation based on confusion matrix.

222 features Random Forest	Completeness of a class	Purity of a class	Contamination of a class
Hippocampus	80%	62%	38%
Not-hippocampus	62%	80%	20%
Efficiency	**70%**		

**Table 11:** For each implemented method, size of selected group, mean Dice index (evaluated using Näive Bayes Classifier), and related *σ* are shown.

Method	Size of selected group	Dice index
Original dataset	315	0.69 ± 0.04
PCA selection	197	0.62 ± 0.07
K-S selection	57	0.67 ± 0.04
Forward selection	36	0.75 ± 0.02
Backward elimination	23	0.75 ± 0.02
Random Forest	222	0.69 ± 0.04
